# Association of publication record and independent NIH funding

**DOI:** 10.1371/journal.pone.0269283

**Published:** 2022-06-30

**Authors:** Kenneth A. Michelson

**Affiliations:** Division of Emergency Medicine, Boston Children’s Hospital, Boston, MA, United States of America; University of Luxembourg, LUXEMBOURG

## Abstract

**Background:**

Publications may be a modifiable factor toward research project grant (RPG) funding decisions, the objective was to determine the association of publication record with later RPG receipt.

**Methods:**

This was a retrospective cohort study of recipients of K01, K08, or K23 US career development awards (CDAs) starting from 2000–2015. Exposures were CDA awardees’ first-, middle-, and last-author publication counts, and the quartile of awardees’ highest and mean publication impact factors. The independent association of each exposure with time to RPG (R01 or equivalent) was determined using a Cox model, after adjustment for CDA type, awardee change in institution, and institutional CDA count. The proportion of CDA recipients with later independent funding was also determined by publication count.

**Results:**

Among 6744 CDA awardees, 3943 obtained an RPG. The median time to RPG was 5.6 years (interquartile range 4.2–7.5). The number of first-authorships was associated with a shorter time to RPG (1–4 versus 0: hazard ratio [HR] 1.22, 95% confidence interval [CI] 1.10–1.36; 5–9: 1.59, 95% CI 1.40–1.79; 10–24: 1.78, 95% CI 1.54–2.07; 25+: 2.40, 95% CI 1.61–3.56). Last-authorships were associated with a shorter time to RPG (1–4 versus 0: HR 1.99, 95% CI 1.83–2.16; 5–9: 2.72, 95% CI 2.45–3.03; 10–24: 3.17, 95% CI 2.78–3.62; 25+: 3.12, 95% CI 2.17–4.50). Higher maximum impact factor was associated with a shorter time to RPG (Q2 versus lowest: HR 1.28, 95% CI 1.12–1.46; Q3: 1.45, 95% CI 1.24–1.70; Q4: 1.67, 95% CI 1.39–2.02). Mean impact factor and middle-authorships were not associated with time to RPG. Among 687 CDAs with zero associated first- or last-authorships, 158 (23%) achieved later RPG funding. Among those with at least 10 total first- or last-authorships, 1288/1554 (83%) obtained a later RPG.

**Conclusions:**

A higher number and impact of publications was associated with later independent funding.

## Introduction

US career development awards (CDAs) are a key step toward independent research careers [1]. These awards provide substantial project and salary support to junior investigators, and provide a pathway to develop the skills and experience to lead large projects. Independent research project grants (RPGs) and individual large project grants (R01 awards) are one of the premier means of supporting biomedical research careers, and are therefore a key outcome of CDAs [2]. These large grants sustain researchers and are a critical source of biomedical innovation in the US [3].

Most CDA recipients will go on to obtain independent research funding, but substantial sex [4–6] and race [7–9] disparities in this progression exist. Medical school affiliation, institutional funding track record, citations, and caregiving responsibilities partially mediate these disparities [7, 10]. Additionally, attracting and retaining a younger and more diverse research workforce had become increasingly difficult, as older researchers obtain a larger share of funding and as the total amount of potential funding has grown more slowly than the research workforce [10, 11].

A better understanding of the modifiable characteristics of CDA recipients, such as publication record, that predict later RPG or R01 funding could help provide a blueprint for how CDA recipients might progress to independence.

## Materials and methods

I studied US federal CDAs (K01, K08, or K23) with start dates from 2000–2015, excluding any awardees with a prior RPG. The data source was the NIH RePORTER database, 2000–2020.

The main outcome was the receipt of US federal independent research funding, analyzed as either an R01 award or RPG [1] (DP1, DP2, DP3, DP4, DP5, P01, P42, PN1, PM1, R00, R01, R03, R15, R21, R22, R23, R29, R33, R34, R35, R36, R37, R50, R55, R56, R61, RC1, RC2, RC3, RC4, RF1, RL1, RL2, RL9, RM1, U01, U19, U34, UA5, UC1, UC2, UC3, UC4, UC7, UF1, UG3, UH2, UH3, UH5, UM1, or UM2).

Potential predictors of research funding included CDA type, funder Institute and Center (IC) for ICs with at least 100 CDAs during the study, whether the CDA required resubmission to achieve funding, change of awardee institution during the CDA period, number of CDA-associated first-author papers, number of CDA-associated middle-author papers, number of CDA-associated last-author papers, highest impact factor of the CDA-associated papers’ journals, mean impact factor, awardee institution located in the US, and whether the awardee institution was in the top 20, next 30, or outside the top 50 institutions in number of CDAs. Journal impact factors were drawn from the Web of Science 2019 Journal Citation Reports [12].

I determined the association of each predictor with time to independent research funding. To make this comparison, I constructed Kaplan-Meier survival curves for each type of independent research funding stratified on predictor variables and tested each association using a log rank test. All researchers who did not achieve R01 or RPG funding were censored on December 31, 2020. The independent association of each predictor with later funding was evaluated using a Cox proportional hazard models including all predictors except for funder IC (because there were too many ICs to feasibly include). Finally, to examine the role of publishing and of ICs, the proportion of CDA recipients who went on to independent funding was determined by number of first- and last-authorships, and separately by funder IC.

## Results

I analyzed 6,744 CDAs after excluding 293 (4.2%) because the awardees had a previous RPG. Demographic features of the cohort are displayed in **Table 1**. Most CDAs were either 3–4 years (23.2%) or 5 years (63.3%) in duration.

**Table 1:** Demographic features of career development awards (CDAs) and their recipients between 2000–2015.

Characteristic	n (%)
CDA type	
K01	1876 (27.8)
K08	2672 (39.6)
K23	2196 (32.6)
Institute or Center (%)	
Agency for Healthcare Research and Quality	104 (1.5)
Eunice Kennedy Shriver National Institute of Child Health/Human Dev	348 (5.2)
National Cancer Institute	592 (8.8)
National Center for Research Resources	219 (3.2)
National Eye Institute	122 (1.8)
National Heart, Lung, and Blood Institute	950 (14.1)
National Institute of Allergy and Infectious Diseases	611 (9.1)
National Institute of Arthritis and Musculoskeletal and Skin Diseases	257 (3.8)
National Institute of Diabetes and Digestive and Kidney Diseases	1081 (16.0)
National Institute of General Medical Sciences	99 (1.5)
National Institute of Mental Health	730 (10.8)
National Institute of Neurological Disorders and Stroke	393 (5.8)
National Institute of Nursing Research	98 (1.5)
National Institute on Aging	345 (5.1)
National Institute on Alcohol Abuse and Alcoholism	109 (1.6)
National Institute on Drug Abuse	271 (4.0)
Other Institute or Center	415 (6.2)
Changed institution during CDA	2724 (40.4)
Resubmitted CDA	2296 (34.0)
Quartile of highest journal impact factor of CDA-associated studies	
Q1: 0–5	1668 (24.7)
Q2: 5–9	1749 (25.9)
Q3: 9–17	1667 (24.7)
Q4: 17–292	1660 (24.6)
Quartile of mean journal impact factor of CDA-associated studies	
Q1: 0–3	1657 (24.6)
Q2: 3–5	1665 (24.7)
Q3: 5–7	1706 (25.3)
Q4: 7–75	1716 (25.4)
Number of first-author CDA-associated studies	
0	1151 (17.1)
1–4	3545 (52.6)
5–9	1472 (21.8)
10–24	546 (8.1)
25+	30 (0.4)
Number of last-author CDA-associated studies	
0	2509 (37.2)
1–4	2824 (41.9)
5–9	927 (13.7)
10–24	447 (6.6)
25+	37 (0.5)
Number of middle-author CDA-associated studies	
0	2172 (32.2)
1–4	3003 (44.5)
5–9	943 (14.0)
10–24	535 (7.9)
25+	91 (1.3)
Total number of CDA-associated studies	
0	455 (6.7)
1–4	1993 (29.6)
5–9	1768 (26.2)
10–24	1937 (28.7)
25+	591 (8.8)
Acquired a patent during the CDA period	232 (3.4)
Duration of CDA	
<3	271 (4.0)
3–4	1564 (23.2)
5	4271 (63.3)
>5	638 (9.5)
Primarily affiliation with a US-based institution	172 (2.6)
Rank of institution’s total number of CDAs, 2000–2015	
Top 20	3240 (48.0)
21–50	1666 (24.7)
Not top 50	1838 (27.3)
Obtained supplemental funding during CDA	503 (7.5)
Total CDA cost, median thousand dollars (interquartile range	634 (509, 708)
Obtained R01	3150 (46.7)
Obtained RPG	3943 (58.5)

R01s were obtained after 3,150 (46.7%) CDAs and RPGs after 3,943 (58.5%). The median time to R01 among awardees was 5.6 years (interquartile range [IQR] 4.2–7.5). Among RPG awardees it was 5.0 years (IQR 3.5–6.7). The median time to R01 was 0.9 years longer among 5-year compared with 3-4-year CDA awardees. The median time to RPG was 0.8 years longer among 5-year compared with 3-4-year CDA awardees.

**Fig 1:**
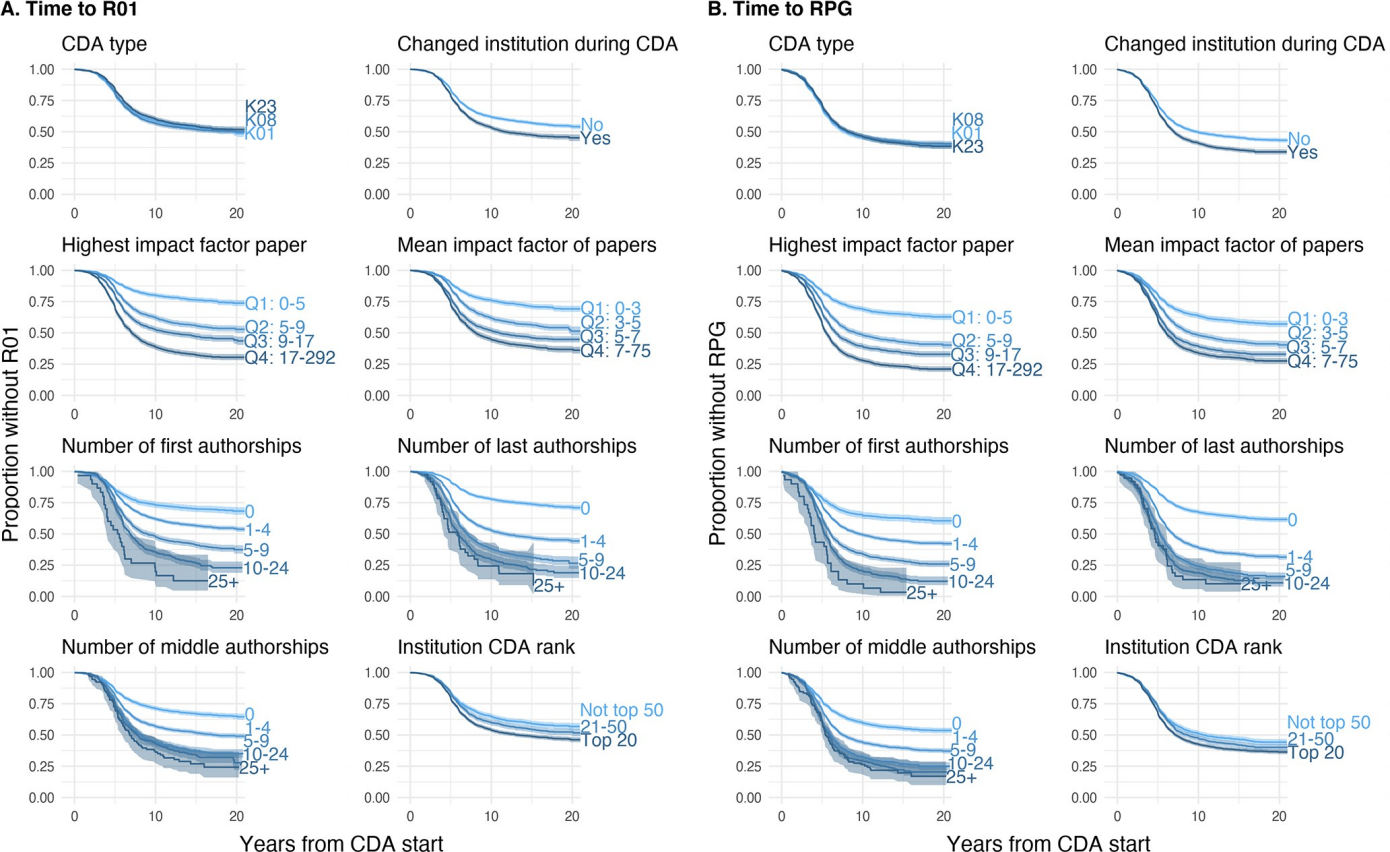
Kaplan-Meier curves depicting time to (A) R01 and (B) RPG award stratified on several characteristics of career development awards.

Time to R01 and time to RPG were each shorter among CDA awardees who changed institution during their CDA, those with higher impact papers, more first, last, or middle authorships associated with the CDA, and among CDAs awarded to top 20 institutions (p<0.0001 for each comparison, **Fig 1**). There was no association between CDA type (K01, K08, or K23) and time to R01 or RPG.

The multivariable Cox regression model demonstrated that each predictor except middle authorships were independently associated with time to R01 or RPG (**Table 2**). For both R01s and RPGs, predictors independently associated with a longer time to independent award were K23, no change of institution during the CDA, lower maximum impact factor of CDA-associated papers, lower mean impact factor of CDA-associated papers, fewer first authorships, fewer last authorships, and affiliation with an institution outside the top 20 CDA-awarded institutions. The strongest predictors were the numbers of first and last authorships.

**Table 2:** Factors associated with time to R01 or RPG acquisition after a career development award (CDA). Hazard ratios (HR) and 95% confidence intervals (CI) were determined using a multivariable Cox proportional hazards approach.

Factor	R01		RPG	
	n (%)	HR (95% CI)	n (%)	HR (95% CI)
CDA type				
K01	873 (46.5)	Ref	1100 (58.6)	Ref
K08	1280 (47.9)	0.92 (0.85, 1.01)	1546 (57.9)	0.90 (0.83, 0.97)
K23	997 (45.4)	0.69 (0.63, 0.76)	1297 (59.1)	0.76 (0.70, 0.82)
Changed institution during CDA				
No	1730 (43.0)	Ref	2197 (54.7)	Ref
Yes	1420 (52.1)	1.10 (1.03, 1.19)	1746 (64.1)	1.08 (1.02, 1.16)
Quartile of highest journal impact factor of CDA-associated studies				
Q1: 0–5	399 (23.9)	Ref	591 (35.4)	Ref
Q2: 5–9	771 (44.1)	1.33 (1.14, 1.55)	997 (57.0)	1.28 (1.12, 1.46)
Q3: 9–17	872 (52.3)	1.46 (1.21, 1.76)	1084 (65.0)	1.45 (1.24, 1.70)
Q4: 17–292	1108 (66.7)	1.78 (1.44, 2.20)	1271 (76.6)	1.67 (1.39, 2.02)
Quartile of mean journal impact factor of CDA-associated studies				
Q1: 0–3	475 (28.7)	Ref	680 (41.0)	Ref
Q2: 3–5	729 (43.8)	1.06 (0.92, 1.21)	946 (56.8)	1.00 (0.89, 1.13)
Q3: 5–7	906 (53.1)	1.18 (1.00, 1.39)	1114 (65.3)	1.07 (0.92, 1.23)
Q4: 7–75	1040 (60.6)	1.42 (1.18, 1.71)	1203 (70.1)	1.24 (1.05, 1.46)
Number of first-author CDA-associated studies				
0	345 (30.0)	Ref	437 (38.0)	Ref
1–4	1531 (43.2)	1.12 (0.99, 1.26)	1967 (55.5)	1.22 (1.10, 1.36)
5–9	857 (58.2)	1.52 (1.32, 1.74)	1048 (71.2)	1.59 (1.40, 1.79)
10–24	391 (71.6)	1.69 (1.44, 1.99)	462 (84.6)	1.78 (1.54, 2.07)
25+	26 (86.7)	2.20 (1.45, 3.35)	29 (96.7)	2.40 (1.61, 3.56)
Number of last-author CDA-associated studies				
0	664 (26.5)	Ref	918 (36.6)	Ref
1–4	1486 (52.6)	2.05 (1.86, 2.25)	1859 (65.8)	1.99 (1.83, 2.16)
5–9	628 (67.7)	2.73 (2.42, 3.07)	748 (80.7)	2.72 (2.45, 3.03)
10–24	341 (76.3)	3.37 (2.91, 3.90)	385 (86.1)	3.17 (2.78, 3.62)
25+	31 (83.8)	3.49 (2.39, 5.10)	33 (89.2)	3.12 (2.17, 4.50)
Number of middle-author CDA-associated studies				
0	720 (33.1)	Ref	972 (44.8)	Ref
1–4	1443 (48.1)	1.07 (0.97, 1.18)	1812 (60.3)	1.04 (0.96, 1.14)
5–9	579 (61.4)	1.16 (1.03, 1.31)	680 (72.1)	1.06 (0.95, 1.19)
10–24	342 (63.9)	1.06 (0.91, 1.22)	406 (75.9)	0.99 (0.87, 1.13)
25+	66 (72.5)	1.05 (0.81, 1.38)	73 (80.2)	0.94 (0.73, 1.21)
Rank of institution’s total number of CDAs, 2000–2015				
Top 20	1662 (51.3)	1.24 (1.14, 1.36)	2006 (61.9)	1.15 (1.06, 1.24)
21–50	755 (45.3)	1.06 (0.95, 1.17)	963 (57.8)	1.02 (0.93, 1.11)
Not top 50	733 (39.9)	Ref	974 (53.0)	Ref

**Fig 2:**
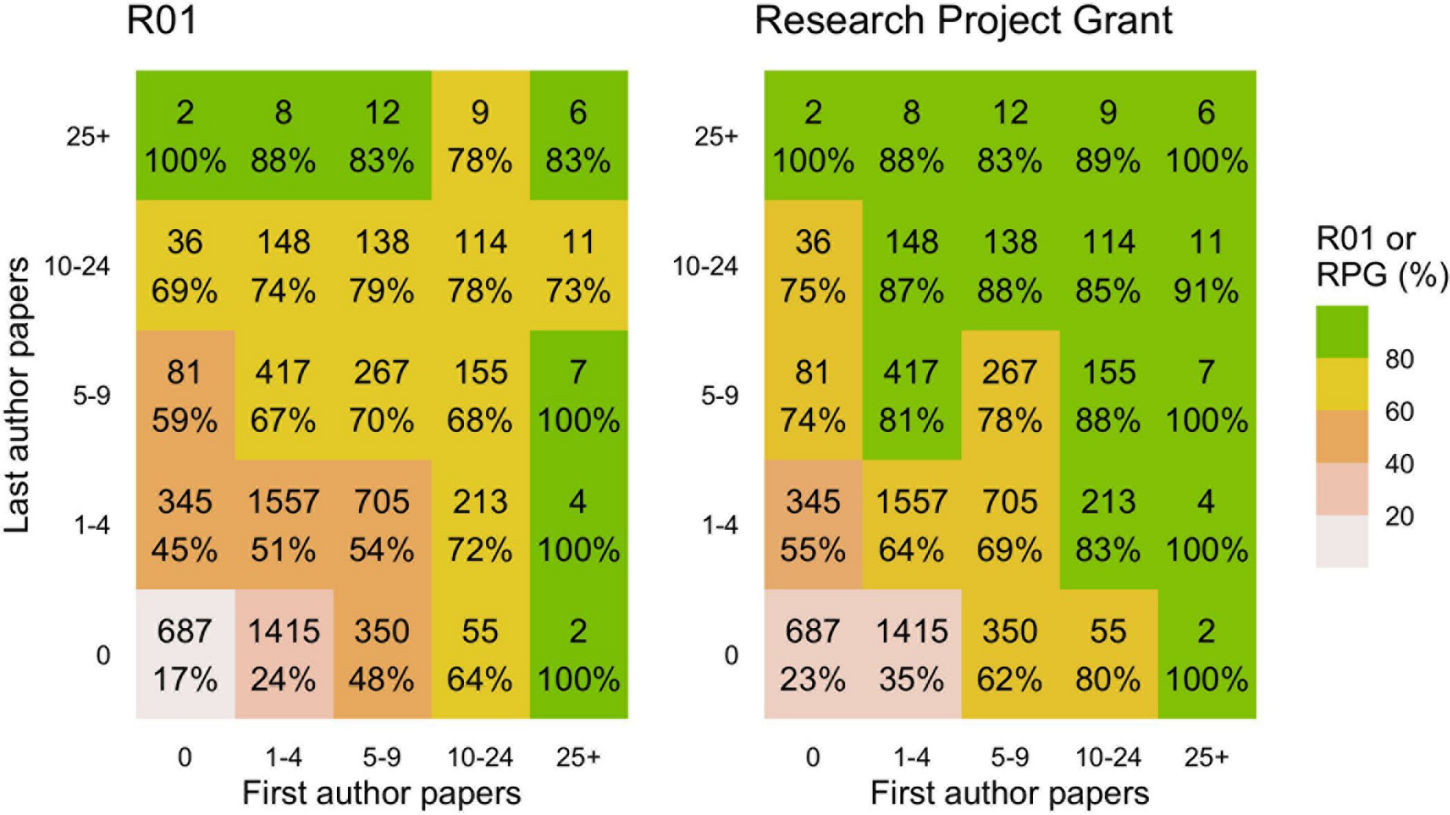
Number and proportion of career development awardees who ultimately received R01 (left) or RPG (right) funding, depending on the number of first or last author papers associated with the career development award.

More CDA-associated first or last authorships substantially improved the proportion of applicants who obtained later R01 or RPG funding (**Fig 2**). Among those with no first or last authorships, 115/687 (17%) obtained a later R01 and 158/687 (23%) obtained a later RPG. By contrast, of those who had at least 10 total first or last authorships, 1108/1554 (71%) obtained a later R01 and 1288/1554 (83%) obtained a later RPG.

**Fig 3:**
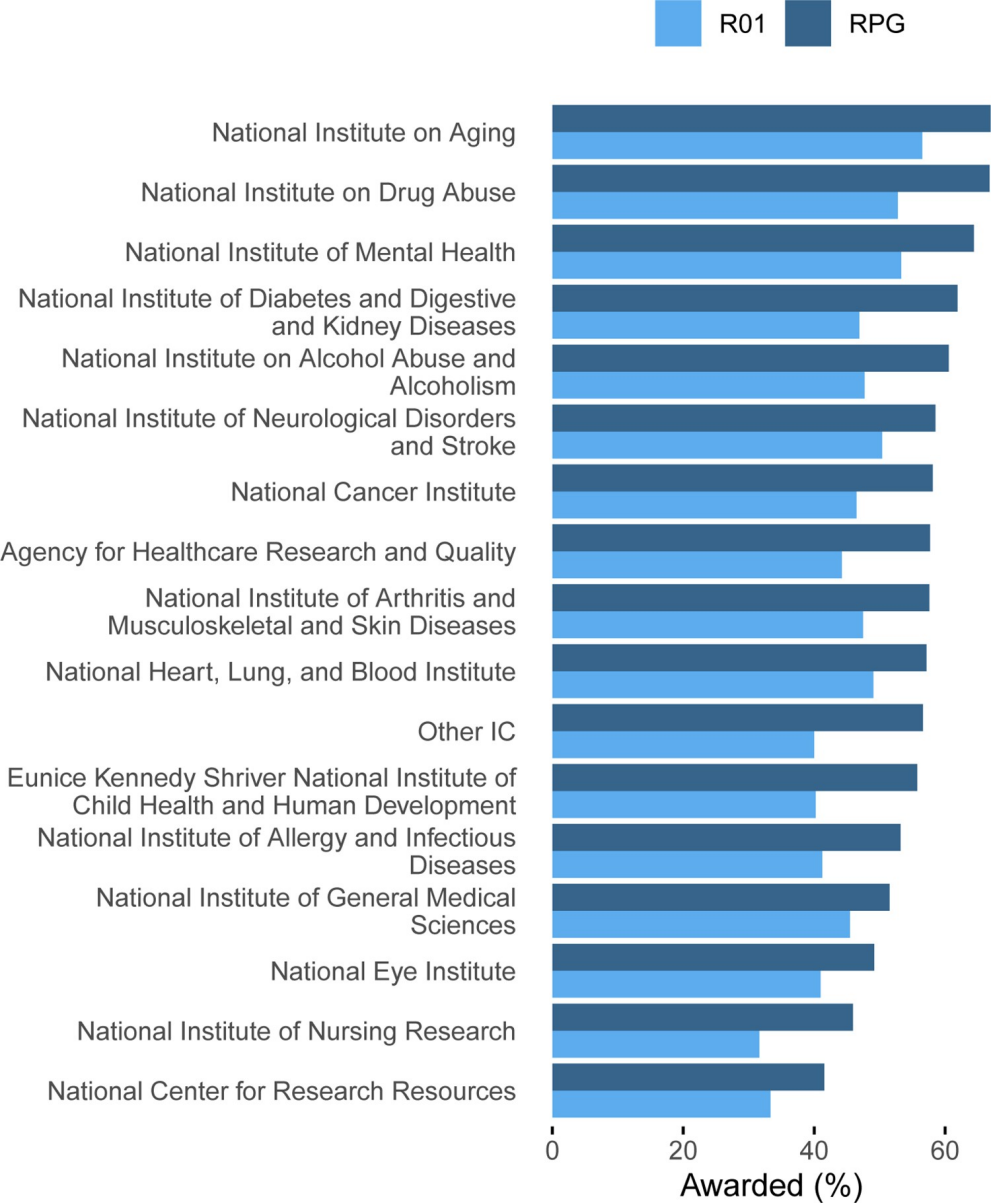
Proportion of career development award (CDA) recipients who received later R01 (grey) or RPG (black) funding, depending on the Institute or Center that funded the CDA.

ICs varied in later funding for CDA recipients (**Fig 3**). The National Institute on Aging had the highest proportion of later R01s (195/345, 57%) and RPGs (231/345, 67%). The National Institute of Nursing Research had the fewest later R01s (31/98, 32%) and the National Center for Research Resources had the fewest later RPGs (91/219, 42%).

## Discussion

Among 6,744 CDA recipients from 2000–2015, approximately half later obtained US research project funding. The number and impact of publications was a very strong predictor of success in later independent funding: approximately one fifth of those without first or last authorships go on to later funding, whereas approximately four fifths with at least 10 first or last authorships go on to later funding.

First and last authorships measure different phenomena [13]. First authorships represent work the author leads, and last authorships represent work the author supervises. Middle authorship frequently reflects contributions to a larger team. Middle authorships were not independently predictive of independent research funding success. Taken together, these findings suggest that CDA recipients with a high number of publications reflecting leadership, mentorship, or both will go on to obtain independent funding. These findings could differ in other nations where funding decisions might rely on other criteria.

This study could not evaluate the reasons that more publications are associated with a shorter time to independent research funding. However, regardless of the actual causes, the implications are clear and reflect the adage “publish or perish.” CDA recipients with few publications are unlikely to obtain US federal funding, while those with many are highly likely to do so. As a result, known disparities in early research experience, topic choice, and first- and last-authorships by race [14, 15] and gender [16] may, at least in part, mediate funding disparities [8] by those characteristics.

Whether a grant applicant’s number of publications is a useful barometer of future research impact is unclear. Highly influential scientists may not be funded when publication count is a key metric [17]. Alternative approaches include funding all applicants, funding applicants randomly, automated approaches, rewarding citizenship, or alternative rubrics for evaluation of promising science [18]. However, any single approach has positive and negative features. Absent major changes in the approach to evaluating research grant applications, authorship is likely to continue to be a meaningful contributor to application success. Institutions and leaders seeking to improve local funding rates and narrow disparities in funding should therefore encourage, foster, and monitor leadership and supervision among junior researchers, particularly those funded by CDAs.

Our study has several limitations. First, our study could not account for changes in funding rates and priorities over the 16 years I analyzed. Second, I could not account for CDA recipients who left the workforce prior to the end of the study period, who would therefore have been ineligible for the outcome of RPG funding. I believe workforce departures would be uncommon since most CDA awardees are in their early career. Finally, I could not directly measure the degree of mediation of publications with disparities, as I did not have access to CDA recipient demographics.

## Conclusions

Among CDA awardees from 2000–2015, first- and last-authorship and impactful publications were strong independent predictors of a shorter time to R01 or RPG funding. Fostering local research productivity could improve independent grant funding success rates among junior researchers. The effect of improving publication counts on disparities in research funding should be undertaken.
